# Early downregulation of hsa-miR-144-3p in serum from drug-naïve Parkinson’s disease patients

**DOI:** 10.1038/s41598-022-05227-6

**Published:** 2022-01-25

**Authors:** Elisa Zago, Alessandra Dal Molin, Giovanna Maria Dimitri, Luciano Xumerle, Chiara Pirazzini, Maria Giulia Bacalini, Maria Giovanna Maturo, Tiago Azevedo, Simeon Spasov, Pilar Gómez-Garre, María Teresa Periñán, Silvia Jesús, Luca Baldelli, Luisa Sambati, Giovanna Calandra-Buonaura, Paolo Garagnani, Federica Provini, Pietro Cortelli, Pablo Mir, Claudia Trenkwalder, Brit Mollenhauer, Claudio Franceschi, Pietro Liò, Christine Nardini, PROPAG-AGEING Consortium, Astrid Adarmes-Gómez, Tiago Azevedo, Maria Giulia Bacalini, Luca Baldelli, Anna Bartoletti-Stella, Kailash P. Bhatia, Bonilla-Toribio Marta, Claudia Boninsegna, Marcella Broli, Buiza-Rueda Dolores, Giovanna Calandra-Buonaura, Sabina Capellari, Mario Carrión-Claro, Rosalia Cilea, Robert Clayton, Pietro Cortelli, Alessandra Dal Molin, Silvia De Luca, Patrizia De Massis, Giovanna Maria Dimitri, Ivan Doykov, Rocio Escuela-Martin, Giovanni Fabbri, Claudio Franceschi, Anna Gabellini, Paolo Garagnani, Cristina Giuliani, Pilar Gómez-Garre, Pietro Guaraldi, Sara Hägg, Jenny Hällqvist, Claire Halsband, Wendy Heywood, Henry Houlden, Ismae Huertas, Silvia Jesús, Juulia Jylhävä, Miguel A. Labrador-Espinosa, Cristina Licari, Pietro Liò, Claudio Luchinat, Daniel Macias, Stefania Macrì, Francesca Magrinelli, Juan Francisco Martín Rodríguez, Delledonne Massimo, Maria Giovanna Maturo, Giacomo Mengozzi, Gaia Meoni, Francesco Mignani, Maddalena Milazzo, Kevin Mills, Pablo Mir, Brit Mollenhauer, Christine Nardini, Stefania Alessandra Nassetti, Nancy L. Pedersen, Maria Teresa Periñán-Tocino, Chiara Pirazzini, Federica Provini, Francesco Ravaioli, Claudia Sala, Luisa Sambati, Cesa Lorella Maria Scaglione, Sebastian Schade, Sebastian Schreglmann, Simeon Spasov, Stephen Strom, Cristina Tejera-Parrado, Leonardo Tenori, Claudia Trenkwalder, Paola Turano, Franco Valzania, Rosario Vigo Ortega, Dylan Williams, Luciano Xumerle, Elisa Zago

**Affiliations:** 1 Personal Genomics S.R.L., Verona, Italy; 2 Computer Laboratory, Department of Computer Science and Technology, University of Cambridge, Cambridge, UK; 3 Department of Biomedical and NeuroMotor Sciences (DiBiNeM), University of Bologna, Bologna, Italy; 4 Department of Biotechnological and Applied Clinical Sciences, University of L’Aquila, L’Aquila, Italy; 5 Unidad de Trastornos del Movimiento, Servicio de Neurología y Neurofisiología Clínica, Instituto de Biomedicina de Sevilla, Hospital Universitario Virgen del Rocío/CSIC/Universidad de Sevilla, Seville, Spain; 6 Centro de Investigación Biomédica en Red Sobre Enfermedades Neurodegenerativas (CIBERNED), Madrid, Spain; 7 IRCCS Istituto delle Scienze Neurologiche di Bologna, Bologna, Italy; 8 Department of Experimental, Diagnostic, and Specialty Medicine (DIMES), University of Bologna, Bologna, Italy; 9 Clinical Chemistry, Department of Laboratory Medicine, Karolinska Institutet at Huddinge University Hospital, Stockholm, Sweden; 10 Alma Mater Research Institute on Global Challenges and Climate Change (Alma Climate), University of Bologna, Bologna, Italy; 11 Departamento de Medicina, Facultad de Medicina, Universidad de Sevilla, Seville, Spain; 12 Paracelsus-Elena-Klinik, Kliniktstrasse 16, 34128 Kassel, Germany; 13 Department of Neurosurgery, University Medical Center Göttingen, Göttingen, Germany; 14 Department of Neurology, University Medical Center Göttingen, Göttingen, Germany; 15 Institute of Information Technologies, Mathematics and Mechanics, Lobachevsky University, Nizhny Novgorod, Russia; 16 Consiglio Nazionale delle Ricerche, Istituto per le Applicazioni del Calcolo “Mauro Picone”, 00185 Rome, Italy; 17 Hospital Universitario Virgen del Rocío/CSIC/Universidad de Sevilla, Unidad de Trastornos del Movimiento, Servicio de Neurología y Neurofisiología Clínica, Instituto de Biomedicina de Sevilla, Seville, Spain; 18 Department of Computer Science and Technology, University of Cambridge, Cambridge, UK; 19 Department of Clinical and Movement Neurosciences, Queen Square Institute of Neurology, University College London, London, UK; 20 Centre for Inborn Errors of Metabolism, UCL Institute of Child Health Library, London, UK; 21 Neurology Unit, Medical Oncological Department, S. Maria Della Scaletta Hospital, 40026 Imola (BO), Italy; 22 Azienda Unità Sanitaria Locale di Bologna, ASL Bologna, Bologna, Italy; 23 Laboratory of Systems Medicine of Healthy Aging and Department of Applied Mathematics, Lobachevsky University, Nizhny Novgorod, Russia; 24 Department of Biological, Geological, and Environmental Sciences (BiGeA), Laboratory of Molecular Anthropology and Centre for Genome Biology, University of Bologna, Bologna, Italy; 25 School of Anthropology and Museum Ethnography, University of Oxford, Oxford, UK; 26 Department of Medical Epidemiology and Biostatistics, Karolinska Institutet, Stockholm, Sweden; 27 Department of Clinical Neurophysiology, University Medical Center Göttingen, Göttingen, Germany; 28 Department of Gerontopsychiatry, Rhein-Mosel-Fachklinik Andernach, Andernach, Germany; 29 NIHR Great Ormond Street Biomedical Research Centre, Great Ormond Street Hospital and UCL Great Ormond Street Institute of Child Health, London, UK; 30 Department of Neuromuscular Disorders, UCL Queen Square Institute of Neurology, London, WC1N 3BG UK; 31 CERM, University of Florence, Via Luigi Sacconi 6, Sesto Fiorentino, 50019 Florence, Italy; 32 Department of Chemistry “Ugo Schiff”, University of Florence, Via della Lastruccia 3-13, Sesto Fiorentino, 50019 Florence, Italy; 33 Casa di Cura Villa Baruzziana, Bologna, Italy; 34 Department of Neurosciences, Biomedicine and Movement Sciences, University of Verona, Verona, Italy; 35 Department of Biotechnology, University of Verona, Strada Le Grazie 15, 37134 Verona, Italy; 36 Giotto Biotech Srl, Florence, Italy; 37 Paracelsus-Elena-Klinik, Kassel, Germany; 38 Department of Neurology, University Medical Centre Goettingen, Goettingen, Germany; 39 Istituto per le Applicazioni del Calcolo Mauro Picone, CNR, Via dei Taurini, 19, Rome, Italy; 40 Department of Physics and Astronomy, University of Bologna, Viale Berti Pichat 6/2, Bologna, Italy; 41 Department of Laboratory Medicine, Karolinska Institute and Karolinska Universitetssjukhuset, 171 76 Stockholm, Sweden; 42 Consorzio Interuniversitario Risonanze Magnetiche di Metalloproteine (CIRMMP), Florence, Italy; 43 Neurology Unit, Neuromotor & Rehabilitation Department, Azienda USL-IRCCS di Reggio Emilia, Viale Risorgimento 80, 42123 Reggio Emilia, Italy

**Keywords:** Biological techniques, Computational biology and bioinformatics, Immunology, Molecular biology, Medical research, Neurology

## Abstract

Advanced age represents one of the major risk factors for Parkinson’s Disease. Recent biomedical studies posit a role for microRNAs, also known to be remodelled during ageing. However, the relationship between microRNA remodelling and ageing in Parkinson’s Disease, has not been fully elucidated. Therefore, the aim of the present study is to unravel the relevance of microRNAs as biomarkers of Parkinson’s Disease within the ageing framework. We employed Next Generation Sequencing to profile serum microRNAs from samples informative for Parkinson’s Disease (recently diagnosed, drug-naïve) and healthy ageing (centenarians) plus healthy controls, age-matched with Parkinson’s Disease patients. Potential microRNA candidates markers, emerging from the combination of differential expression and network analyses, were further validated in an independent cohort including both drug-naïve and advanced Parkinson’s Disease patients, and healthy siblings of Parkinson’s Disease patients at higher genetic risk for developing the disease. While we did not find evidences of microRNAs co-regulated in Parkinson’s Disease and ageing, we report that hsa-miR-144-3p is consistently down-regulated in early Parkinson’s Disease patients. Moreover, interestingly, functional analysis revealed that hsa-miR-144-3p is involved in the regulation of coagulation, a process known to be altered in Parkinson’s Disease. Our results consistently show the down-regulation of hsa-mir144-3p in early Parkinson’s Disease, robustly confirmed across a variety of analytical and experimental analyses. These promising results ask for further research to unveil the functional details of the involvement of hsa-mir144-3p in Parkinson’s Disease.

## Introduction

Advanced age is the primary risk factor for the development of Parkinson’s Disease (PD)^1^. As a consequence, ageing represents a crucial variable to understand PD pathogenesis, to identify early biomarkers of the disease and to promote innovative therapies^2^. This is the perspective and the central rationale behind the project PROPAG-AGEING (“*The continuum between healthy ageing and idiopathic Parkinson Disease within a propagation perspective of inflammation and damage: the search for new diagnostic, prognostic and therapeutic targets*”), set up to elucidate the contribution of ageing to PD^3^. PROPAG-AGEING envisages a multi-omic characterization of peripheral biospecimens of PD patients (both de novo, i.e. dopaminergic drug-naïve and *advanced*, i.e. under long-standing dopaminergic drug therapy regimen) in comparison with healthy controls (representative of the general population), and with a cohort of centenarians. This latter group has experienced a particularly successful ageing, likely because of these individuals’ ability to adapt and remodel in response to age-related changes. Centenarians, in fact, reached advanced age without showing clinical signs of motor disability, and can therefore be referred as "super-controls”. As previously reported^4,5^, the strategy to consider a continuum of phenotypes (advanced stage patients, early stage patients, controls and super-controls) enhances the possibility to identify biomarkers and to understand the pathogenesis of diseases. Furthermore, PROPAG-AGEING has recruited a cohort of siblings of PD patients that includes unaffected subjects having a higher risk for developing PD compared to the general population^3^.

While the project explores a variety of omic layers, the present work offers insights on the significance and role of circulating serum microRNAs (miRNAs). miRNAs are a large family of small non-coding RNA that act as post-transcriptional modifiers of gene expression, now recognized as important regulators of lifespan because of their effects on several crucial aging pathways^6^. Moreover, circulating miRNAs in serum or plasma represent potential non-invasive biomarkers of an individual’s current health status^7^.

The study of miRNAs in ageing and PD is not a premiere. Literature focused on the study of miRNAs in serum from subjects of different age already identified a panel of miRNAs that change in strong relationship with the aging process^7,8^. In particular, miRNA profiles have been characterized also in mononuclear cells from young people, octogenarians and centenarians. For the latter, a general miRNAs up-regulation correlated with age has been observed, a fact that can be partly referred to the ability of centenarians to maintain the miRNA biogenesis pathway active^9^. Such analyses also revealed that centenarians show a peculiar miRNA profile closer to youngster than to octogenarians, supporting the hypothesis that centenarians follow a healthy-ageing trajectory^10^.

Alterations of miRNA profiles have also been reported in pathological conditions, including neurodegenerative diseases^11,12^. In fact, the relevance of miRNAs in the biology of PD has already been proven in several studies^11^ and some reports evaluated the levels of miRNA in serum and plasma as potential biomarkers of the disease (reviewed in Refs.^13–15^). However, as most of these studies include samples from advanced patients, the identified miRNA signatures could be the result of drug treatment and/or disease progression. Until now, in fact, only few reports considered de novo PD patients. While, until recently, no significant differences between drug-naïve patients and controls were found^16,17^, possibly owing to the small sample size, recently, Patil et al.^18^ used microarrays to profile a large cohort of de novo samples and identified a miRNA serum signature specific to PD.

In light of this promising landscape, the present work aims at exploring the relevance of miRNAs as biomarkers of PD within the ageing framework.

## Methods

### Materials: serum samples

Serum samples from centenarians (CENT), de novo patients (dnPD), controls (CTR), advanced patients (adPD) and patients’ siblings (PDsibs) were collected at three different Collection Sites: the University of Bologna (UNIBO; 27 CENT and 19 CTR), the University of Goettingen (GOE; 205 dnPD, 12 adPD, 58 CTR) and the Servicio Andaluz de Salud (SAS; 48 PDsibs). Samples were used in two distinct phases (see details below): *discovery* to identify potential biomarkers and *validation* to assess the robustness of the findings. Samples from UNIBO were recruited in the framework of a study approved by the local Ethical Committee (S. Orsola Hospital—University of Bologna; Prot. n. 2006061707, amendment 08/11/2011). Informed consent was obtained from all subjects involved in the study. The samples from GOE used in the discovery phase (61 dnPD and 58 CTR) were part of the DeNoPa cohort, a longitudinal study previously described^19,20^ retaining only baseline samples. The samples from GOE used in the biological validation were part of the independent, cross-sectional Kassel cohort more recently collected^21^. Patients enrolled in this study were clinically phenotyped and diagnosed as PD according to UK Brain Bank Criteria^22^ before collection of samples. Phenotyping included Unified PD Rating Scale, 1.5 Tesla magnetic resonance imaging, quantitative levodopa testing as published^23^, smell identification test, Mini Mental Status Examination (MMSE) and further cognitive testing, as well as video-supported polysomnography to determine REM sleep behaviour disorder in a subset of patients. Subjects with marked vascular lesions in MRI or with normal pressure hydrocephalus by MRI were excluded.

Siblings of patients with a diagnosis of sporadic idiopathic PD (PDsibs) were recruited over 20 months between September 2016 and January 2019 by Servicio Andaluz de Salud (Spain). The study was approved by the local Ethics Committee (ethical committee no. of approval 2014/PI173 of September 2016).

Serum miRNA were extracted using the miRNeasy Serum/Plasma Advanced kit (Qiagen) according to the manufacturer's instructions. All experiments were performed in accordance with relevant guidelines and regulations.

### Library preparation and miRNA sequencing

Libraries for miRNA sequencing (NGS data) were prepared using QIAseq miRNA Library kit (Qiagen) following manufacturer’s instructions. Labchip DNA High Sensitivity assay (Perkin Elmer) was used to analyse miRNA libraries, further quantified using the Qubit 2.0 Fluorometer (ThermoFisher). Libraries were sequenced with read length of 75 bp on a NextSeq 500 sequencer (Illumina). The total sequencing reads obtained per sample were 10.83 million, on average. The data have been deposited in NCBI's Gene Expression Omnibus^24^ and are accessible through GEO Series accession number GSE180193 (https://www.ncbi.nlm.nih.gov/geo/query/acc.cgi?acc=GSE180193).

### Bioinformatic pre-processing

The sequencing data were processed using a bioinformatic pipeline produced *in house* across three main steps: (i) pre-processing, (ii) differential expression analysis, (iii) functional annotation. The trimming of miRNA sequencing adapters and selection of sequences corresponding to mature miRNAs, was performed using Cutadapt v1.18^25^. The filtered reads were then aligned on the miRbase human hairpin database release 22.1^26^ and on Exiseq NGS Spike-In sequences using SHRiMP v2.2.3^27^. Alignment was performed from fastq files interpreting quality values in fastq input as PHRED + (parameter “-qv-offset 33”), mode “mirna”, and “-strata” option, to output the highest scoring mappings for any given read. The BEDTools suite v2.27.0^28^ was used to select overlapping mappings on miRNA hairpins with mature miRNAs. qPCR duplicates filtering and UMI (Unique Molecular Identifiers) counts summarization was performed using a custom script to process same sequence and same UMI (Unique Molecular Identifier) sets of reads and to return a single read for each set.

The average number of reads mapped to mature miRNAs was between 2 and 3 million. The average number of miRNAs detected per sample was between 524 and 602. The final raw counts matrix had 2578 expressed miRNAs (UMI count > 0 in at least one sample) and 165 samples.

### Differential expression

The miRNAs expression counts were normalized using R package DESeq2 v1.22.1^29^. Principal component analysis (PCA) was performed using as input the VST (Variance Stabilizing Transformation) counts and plots were examined for quality check. Differential expression analysis was performed using the Wald test, and miRNAs with adjusted p-value below 0.05 were selected as differentially expressed (DEMs).

### Validation and biomarkers selection

**Figure 1:**
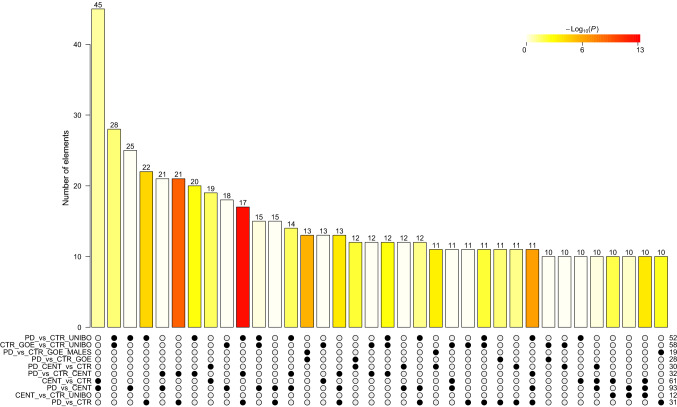
Intersection plot reporting the number of shared DEMs. The matrix at the bottom represents a table whose rows are the DESeq2 contrasts (names on the left and size on the right end), and whose columns represent the results of selected contrasts’ intersections (solid black circle). The sizes of each intersection is visually depicted by the bars color-coded by statistical significance (p-value), and sizes reported on top. The minimum number of shared DEMs in the plot is 10. The plot was created using the R package *upset v. 1.4.0*^48^ (https://cran.r-project.org/web/packages/UpSetR/index.html), R software version 3.5.1.

Technical validation of NGS results was achieved by qPCR. To this purpose, a subset of DEMs was selected by ranking adjusted p-values and filtering out average normalized expression below 5 counts (according to the qPCR plates supplier recommendations). Additional filtering was done considering the most significant overlaps among the already performed comparisons (Fig. 1). MiRNAs were selected among contrasts with higher statistical significance resulting from the DEMs intersection analysis: candidates were selected either when (i) shared between the most statistically significant contrasts or (ii) being the most significant and unique to a contrast. The final set of miRNA targets included also two miRNAs selected from the literature named “inflamma-miR”^30,31^. All details can be found in Supplementary Table S2.

### Quantitative RT-PCR analysis

Technical validation on NGS data was performed using 12 plates for measuring the expression of 28 miRNA targets, 3 endogenous controls and 1 exogenous control (cel-miR-39-3p). For each plate 6 samples were processed: 1 CENT sample (UNIBO), 1 CTR sample (UNIBO), 2 CTR samples (GOE), 2 dnPD samples (GOE), to avoid batch effects. The cDNA was synthesized using TaqMan Advanced miRNA cDNA Synthesis according to the manufacturer’s instructions. Further, 1 µl of spike-in cel-miR-39-3p 5 pM was added to poly(A) tailing reaction to check for reverse transcription efficiency. The qPCR validation was performed using pre-plated TaqMan Advanced miRNA Assays (ThermoFisher) and Taqman Fast ADVANCED Master MIX in a QuantStudio5 instrument. Initially, three miRNA normalizers were selected for validation by analysing four NGS samples (2 dnPD samples, 1 CTR from GOE and 1 CTR from UNIBO) on two custom-made ready-to-use plates with 30 miRNAs as endogenous controls (ThermoFisher). The selection of the three endogenous controls was made both on the Ct range and on the NGS analysis results (stable across conditions, as any variation in their expression may obscure real changes and produce artefacts in expression). After data review, miR-16-5p, miR-93-5p and miR-186-5p were selected as endogenous controls for the normalization. Real-time qPCR was performed in duplicate.

Biological validation on the independent cohorts was performed using in total 17 plates for measuring expression of 3 miRNA targets, 1 endogenous control (miR-186-5p) and 1 exogenous control (cel-miR-39-3p). The cDNA was synthesized using TaqMan Advanced miRNA cDNA Synthesis according to the manufacturer’s instructions. 1 µl of spike-in cel-miR-39-3p 5 pM was added to poly(A) tailing reaction. The qPCR reaction was performed for miR-144-3p, miR-150-5p and miR-215-5p using Advanced miRNA Assay with Taqman Fast ADVANCED Master MIX in a QuantStudio3 instrument, again, qPCR was performed in duplicate.

The qPCR differential expression analysis was performed using the Applied Biosystems™ Analysis Software web application (apps.thermofisher.com), Relative Quantitation Analysis Module (2020.2.1-Q2-20-build4 of 2020-04-27) in Singleplex mode, Version 1.1 and the CT method. The endogenous controls were used for normalization and the T-test statistic for computing p-values, adjusted with Benjamin-Hochberg statistical method. By default, significant differential expression is reported by the software when the observed RQ (Fold Change) is > 2 or < 0.5 and the corrected p-value is < 0.05.

### ARACNE and network reconstruction

The miRNAs interaction matrix was reconstructed using the ARACNE network imputation tool^32^. The network reconstruction approach is part of a wider set of gene network inference tools, based on information-theory related methods^33^. First, the mutual information ${M}_{ij}$ is computed between samples, using a Gaussian kernel methodology^32–34^. Second, the mutual information matrix is thresholded, and all edges for which the condition of genes being mutually independent cannot be ruled out, are discarded from the network. Then, a Data Processing Inequality (DPI) is applied, discarding the weakest edges. In particular we used the Parmigene R (https://cran.r-project.org/web/packages/parmigene/index.html) implementation of the ARACNE reconstruction, with the multiplicative factor. More specifically, given three nodes $i,j$ and $k$, the regulatory network reconstruction removes the weakest edge according to the relationship:

$$M\left(i,j\right)<M\left(j,k\right)\times \left(1-\tau \right)$$

and

$$M\left(i,j\right)<M\left(i,k\right)\times \left(1-\tau \right),$$

where $0<1-\tau <1$ is a multiplicative factor. In our experiments the value of $\tau$ was set tot the default value of 0.15. The matrix of mutual interaction reconstructed using the ARACNE procedure was then used as the adjacency matrix of our network. Finally, communities of miRNAs were identified, through the Louvain Community detection method, described in the following section.

#### Louvain community detection

To perform miRNAs networks clustering, we applied the Louvain Community detection method. The algorithm was first published in 2008^35^, and it was designed to work with large-scale networks, proving to be computationally efficient in time and space for the identification of communities. The number of clusters is automatically determined by the optimization procedure and the algorithm consists in the optimization of an indicator, the modularity, which determines how internally tight are the clusters identified in the iterative community detection procedure. At each iteration the gain in modularity is computed and the optimization process ends when no more improvement is obtained by the reassignment of the elements to a new clustering configuration. The $\gamma$ resolution parameter was set to the default value of 1 in our experiments^35^.

### Target analysis and functional annotation

MiRTarBase v7.0^36–38^ was used to annotate experimentally validated miRNA–mRNA target interactions (MTIs) of the selected miRNA targets. The MTIs were integrated with information contained in miRWalk v2.0^39^ and a database of experiment-supported miRNA-disease association (HMDD v3.2^40^). Functional annotation of the targets was also performed with DAVID Functional Annotation Tool v6.8^41^, to highlight the most relevant functional terms.

MicroRNA-target enrichment, functional enrichment and network-based analysis have been performed using MIENTURNET^42^. For the miRNA-target enrichment analysis, the threshold of minimum number of miRNA-target interactions was set to 2 and the threshold for the adjusted p-value (FDR) was set to 0.05. The miRNA-target interactions database used was miRTarBase website Release 7.0, using only interactions with *Strong* evidence. The plots of miRNAs augmented KEGG pathways from miRNA-targets interactions (miRTarBase v7.0) were generated using the *integrate_mir* function of R package mirIntegrator^43^.

### Ethics approval and consent to participate

The human samples derive from existing multi-centre cohorts collected by the PROPAG-AGEING Consortium partners before the PROPAG-AGEING project, in the framework of other national and international projects.

## Results

### Design of the study

To uncover the complex biology shared by ageing and PD, we first performed miRNA-seq analyses on serum samples from recently diagnosed, *drug-naive* PD patients (dnPD) and age- and sex-matched controls (CTR)^19,20^ and centenarians (CENT) (Table 1, *discovery* cohort). Standard analyses to identify differentially expressed miRNAs^44–47^ were followed by network modelling^32^. Network reconstruction has, in fact, been proven to be a valid tool to model interactions between miRNAs, and several studies have shown the importance of analysing miRNA–miRNA networks to deepen the understanding of phenomena, beyond the identification of differential signatures. Further, miRNAs emerging from these analyses as biomarkers of PD and/or ageing were validated by qPCR, comparing the CTRs from the discovery phase with an independent *validation* cohort including additional dnPD, advanced PD (adPD) and siblings of PD patients (PDsibs) (Table 3).

**Table 1:** Samples of the discovery cohort processed by Next Generation Sequencing.

Collection site	Phenotypic group	Number of samples	Age (mean ± Sd)	Gender (M/F)
GOE	dnPD	61	66 ± 8.2	37/24
GOE	CTR	58	65 ± 8.8	35/23
UNIBO	CENT	27	105 ± 3.5	5/22
UNIBO	CTR	19	68 ± 7.9	6/13

The healthy controls cohort (CTR) consists of 77 samples, 58 of which belonging to the University of Goettingen (GOE) and the remaining 19 to the University of Bologna (UNIBO). The dnPD samples all belong to the GOE site. The 27 centenarians’ samples (CENT) are from UNIBO.

Finally, to complete the study, advanced functional analyses were further run with an array of state-of-the-art tools for the study in silico of miRNAs–mRNA target interactions and functional enrichment—miRWalk^39^; HMDD^40^; miRTarBase^36–38^; MIENTURNET^42^; MiRIntegrator^43^.

### Discovery cohort: unravelling ageing and neurodegenerative facets of PD

#### miRNA-seq differential expression analysis and RT-qPCR validation

The discovery cohort (Table 1) includes 165 serum samples collected in 2 recruiting centers (GOE and UNIBO). GOE samples include 61 dnPDs and 58 age- and sex-matched CTRs, while UNIBO samples include 19 CTRs and 27 CENTs.

The comparisons (a.k.a. *contrasts*) were run to identify differentially expressed miRNAs (DEMs, adjusted p-values < 0.05 and average expression levels > 5). In this analysis, both dnPD and CENT were compared with CTR, considering UNIBO and GOE samples independently (CTR_UNIBO, CTR_GOE, respectively) and combined (CTR_ALL). Centenarians were analysed, as super-controls, in comparison with dnPD samples and CTR. The total number of non-redundant DEMs identified from all comparisons is 169 (Supplementary Table S1), with the largest intersection being 45 DEMs in contrasts CENT vs CTR and dnPD vs CENT, and the most statistically significant ones being dnPD vs (CTR + CENT) and dnPD vs CTR (UNIBO), sharing 17 DEMs (Fig. 1). The analysis then focused on DEMs that were either shared across the maximum number of contrasts or on DEMs that were specific to one contrast only (i.e. specific to a phenotype).

Finally, ranked DEMs were assessed against the literature (see “Methods”), leading to the identification of 28 candidates (Supplementary Table S2) for further technical validation via qPCR (Supplementary Table S4 for dnPD vs CTR, Supplementary Table S5 for CENT vs CTR and Supplementary Table S6 for dnPD vs CENT) over a selection of patients’ samples previously analysed by NGS (Supplementary Table S3).

From this, *hsa-miR-122-5p* and *hsa-miR-150-5p* result to be significantly down-regulated (corrected p-value < 0.05 and log2(FoldChange) < 1) in CENT with respect to CTR while *hsa-miR-215-5p* was significantly down-regulated both in PD and CENT with respect to CTR. Weaker trends were also observed towards down-regulation in PD with respect to CTR for *hsa-miR-150-5p*, and, in dnPDs with respect to CTR and to CENT for *hsa-miR-144-3p*. In a conservative perspective all four miRNAs were retained for the following network-based in silico analysis.

### In silico validation by network reconstruction

**Figure 2:**
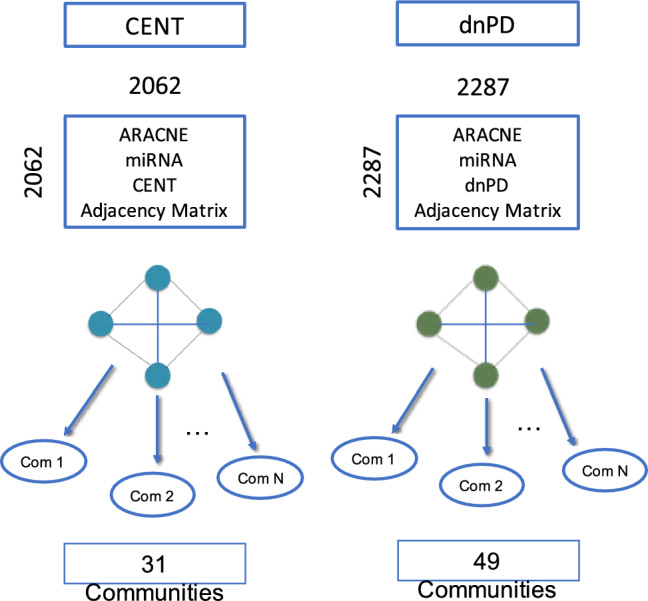
Network analysis. Graphical process of the network analysis implemented for the two cohorts of dnPDs and CENT. Nodes per community are listed by the adjacency matrix box, and the final numbers of communities are shown below each cohort. The figure was created using PowerPoint software Version 16.30, 2019, (https://www.microsoft.com/).

In the effort to further assess the role of these findings, network reconstruction was performed to analyse communities of interacting or co-expressing miRNAs in the two phenotypically relevant cohorts of dnPD and CENT. To reconstruct the networks, two separate adjacency matrices (double entry tables listing a distance between each possible couple of miRNAs, see “Methods” for details) for the dnPD and CENT cohorts were produced using the ARACNE network reconstruction approach^32^. All miRNAs identified in each cohort (i.e. the DESeq2 normalized counts for all samples within a cohort) were used to construct the adjacency matrices, as depicted in Fig. 2. The total number of nodes (miRNAs) and edges (connections between miRNAs, based on their similarity as described in the equation reported in Materials and Methods) obtained for each network is summarized in Supplementary Table S7. The two networks were then partitioned into smaller communities (i.e. clusters) by applying the Louvain community detection algorithm^35^. This enables the extraction of smaller, and putatively more relevant, groups of miRNAs characterized by tighter interactions i.e. similar expression behaviours within each cohort. A total of 31 communities for CENT and 49 communities for dnPDs were obtained (Fig. 2). Additional statistics emerging from the topological analysis of the network were computed to obtain more insight into the relevance of specific nodes: in particular the node degree, i.e. the number of edges departing from a node, is reported in Table 2. The node degree represents a miRNA’s level of connection with other miRNAs, and is therefore a relevant proxy of the molecule’s involvement in a variety of pathways and biological functions^49,50^. The degree can be weighted, or not, by the similarity to the connected miRNA (see “Methods” section for more details). While the unweighted node degree measures the number of connections, the weighted node degree reports the importance of these connections proportionally to the edge number (connectivity of the node) and therefore both are relevant to establish network properties. A full list of the distribution of weighted and unweighted node degrees, can be found in Supplementary Table S8.

**Table 2:** Node degrees statistics of miRNA communities.

miRNA	Weighted node degrees	Unweighted node degrees	Community ID	Community size
**dnPD**				
hsa-miR-144-3p	6.93	30	24	419
hsa-miR-122-5p	8.62	29	24	419
hsa-miR-150-5p	5.52	23	24	419
hsa-miR-215-5p	3.64	18	24	419
**CENT**				
hsa-miR-144-3p	0.85	3	13	35
hsa-miR-122-5p	1.28	4	31	134
hsa-miR-150-5p	0.12	1	20	28
hsa-miR-215-5p	0.58	2	15	97

Columns 1 and 2 show the weighted and unweighted node degrees for each miRNA of interest in the given community. Column 3 reports the community ID, a consequent number, only needed to identify the location of a given miRNA with no other meaning. Column 4 shows the dimension (number of nodes) of each of the community described in Column 3.

Details on the topological distribution of the four miRNAs of interest in the phenotypes under study were achieved when community and differential expression analysis results were integrated. The lists of differentially expressed miRNAs were matched against the communities to identify the maximum overlap between the lists of DEMs and the lists of miRNAs found in the communities.

#### de novo PD

This integration lead, interestingly, to the identification of only one dnPD community containing the four DEMs identified in the NGS analysis and technically validated. **Hsa-miR-150-5p, hsa-miR-215-5p, hsa-miR-144-3p and hsa-miR-122-5p** cluster together in community 24, which in total includes 419 miRNAs. Hsa-miR-144-3p and hsa-miR-122-5p present the highest weighted and unweighted node degrees, respectively, in the community (Table 2). This indicates that these two miRNAs hold the highest amount of pairwise connections and in particular they fall above the upper quartile of the overall weighted degrees distribution of all the nodes in community 24, and between the median and the upper quartile of the unweighted degrees distribution, a proxy for the relevance (ubiquity, conservation^49,50^) of the corresponding molecules.

#### Centenarians

In this case the four miRNAs appear to be assigned to different communities and have low node degrees, with respect to the distribution of all the node degrees in the respective communities as we show in Table 2. The same analysis indicates that the miRNA having the highest weighted and unweighted node degree is **hsa-miR-122-5p** (with both weights falling above the upper quartile of the distribution of the unweighted node degrees and between the median and the upper quartile of the unweighted degrees distribution of the miRNAs in the community).

### Validation in an independent cohort of dnPD, adPD patients and PD siblings

We then focused on the three miRNAs that are statistically significantly differentially expressed in dnPD vs CTR, i.e. hsa-miR-215-5p, hsa-miR-150-5p and hsa-miR-144-3p. A much larger, independent data set including 204 samples (Table 3) was tested via qPCR. Importantly, the validation cohort included, in addition to dnPD samples, also adPDs and PDsibs, absent in the discovery cohort. We used as reference the CTR from UNIBO and GOE already included in the discovery cohort, both independently (CTR_GOE, CTR_BO) and combined (CTR_ALL).

**Table 3:** Samples of the validation cohort.

Collection site	Cohort	Phenotypic group	N. of samples	Age (mean ± Sd)	Gender M/F
GOE	Validation	adPD	12	68 ± 6	9/3
GOE	Validation	dnPD	144	65 ± 12	74/70
SAS	Validation	PDsibs	48	60 ± 12	17/31
UNIBO	Discovery	CTR	12	77 ± 9	2/10
GOE	Discovery	CTR	23	74 ± 8	14/9

The validation was performed on 191 total samples: dnPD and adPD were analysed and compared with CTR from the discovery cohort.

**Figure 3:**
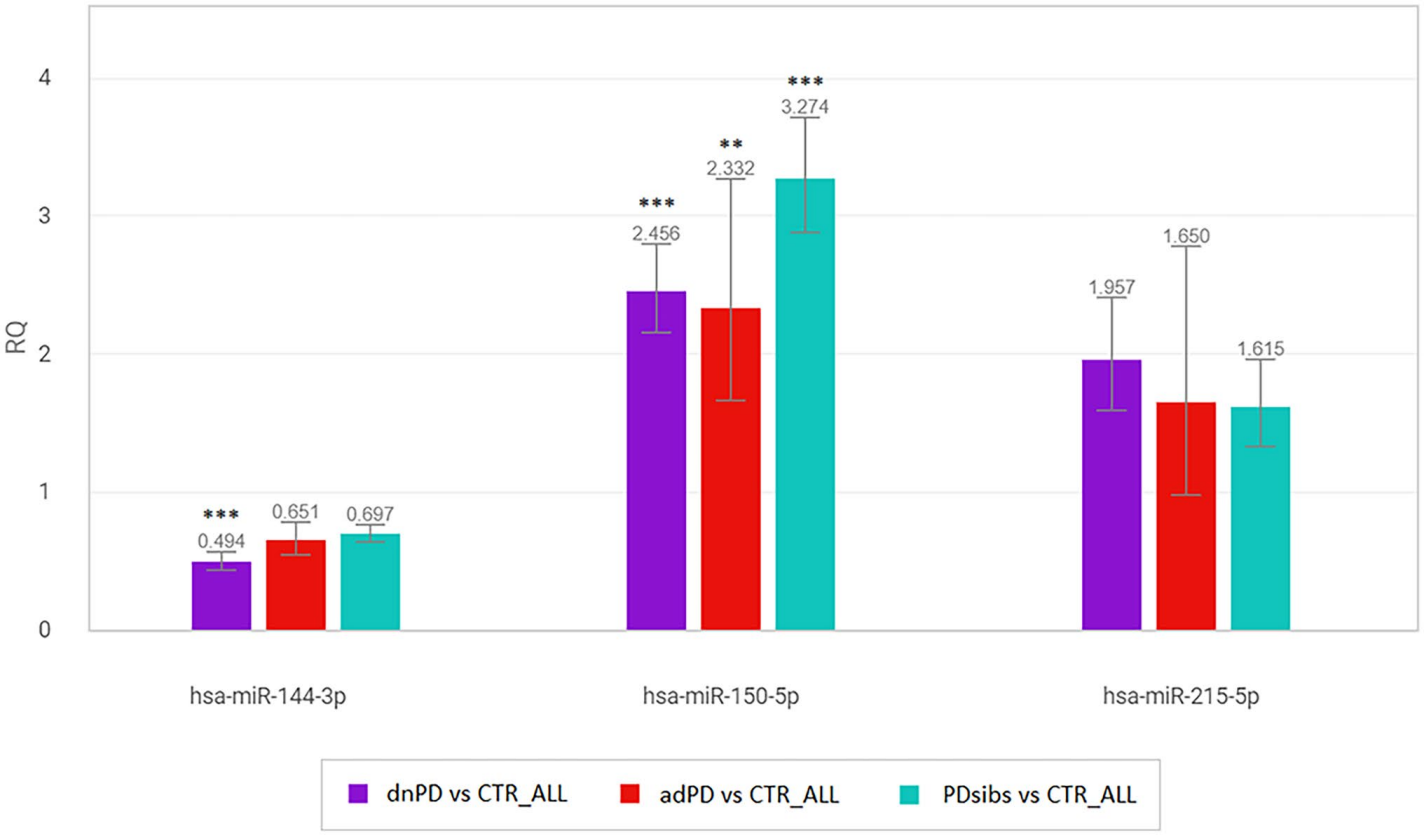
RQ (Relative Quantitation) plot. The plot displays the results of the RT-qPCR relative quantification (dnPDs, adPDs and PDsibs with respect to CTR_ALL) of the gene expression profile of hsa-miR-150-5p, hsa-miR-215-5p and hsa-miR-144-3p, in the validation. Standard asterisks notations indicates **for P-value < 0.01, and Fold Change (RQ) > 2 or < 0.5, and ***for P-value < 0.001 and Fold Change (RQ) > 2 or < 0.5. The image was obtained and downloaded from the software ThermoFisher Applied Biosystems™ Analysis Software web application (apps.thermofisher.com), Relative Quantitation Analysis Module (2020.2.1-Q2-20-build4 of 2020-04-27). The image was afterwards edited with LibreOffice Draw, Libre Office version 5 (https://www.libreoffice.org/).

As shown in Fig. 3 and Table 4, hsa-miR-144-3p appears to be down-regulated in dnPDs with respect to CTR, confirming NGS results and the trend observed in the technical validation (Supplementary Table S9). Interestingly, hsa-miR-144-3p showed a down-regulation trend in adPDs and PDsibs as well, with respect to CTR (Supplementary Tables S10 and S11).

**Table 4:** Results of qPCR differential expression analysis on dnPD, adPD and PDsibs samples vs CTR_ALL.

Test group	Reference group	Target	Log2(FoldChange)	Corrected P-value
dnPD	CTR_ALL	hsa-miR-144-3p	− 1.02	7.31E−05
dnPD	CTR_ALL	hsa-miR-150-5p	1.30	5.44E−07
dnPD	CTR_ALL	hsa-miR-215-5p	0.97	1.17E−04
adPD	CTR_ALL	hsa-miR-144-3p	− 0.62	3.49E−02
adPD	CTR_ALL	hsa-miR-150-5p	1.22	2.00E−03
adPD	CTR_ALL	hsa-miR-215-5p	0.72	2.53E−01
PDsibs	CTR_ALL	hsa-miR-144-3p	− 0.52	3.11E−02
PDsibs	CTR_ALL	hsa-miR-150-5p	1.71	2.71E−10
PDsibs	CTR_ALL	hsa-miR-215-5p	0.69	5.75E−03

The table reports the Corrected P-Value and log2(FoldChange) (threshold for significance set to Corrected P-Value < 0.05 and log2(FoldChange) >|1|).

On the contrary, hsa-miR-150-5p was significantly up-regulated in dnPD, adPD and PDsibs with respect to CTR, and hsa-miR-215-5p showed a similar trend (Supplementary Tables S9, S10 and S11). While confirming the co-regulation of these two miRNAs, this observation did not return the results obtained in the discovery cohort that, therefore, were not considered in the following analyses, focusing exclusively on hsa-miR-144-3p.

### Functional analysis

To gain insights into the potential role of hsa-miR-144-3p in PD, a wide variety of tools and databases for functional inference were exploited as described in the following.

#### miRNA targets: miRTarBASE

Target genes were obtained from a database of experimentally validated miRNA-mRNA target interactions (MTI). MTIs in miRTarBase are annotated as *non-functional*, *strong functional* and *weak functional* based on the technique used for the assay^36–38^. Most of the 211 targets of hsa-miR-144-3p were supported by weak evidence, while 23 genes had a strong functional MTI evidence (3 targets with *non-functional MTIs,* 187 *targets with weak functional MTIs* and 23 *targets with strong functional MTIs*) (Supplementary Table S12).

In addition, hsa-miR-144-3p has been functionally annotated using HMDD3.2^40^, a database of miRNA-disease association and causation obtained using MDCAP (MiRNA-Disease Causal Association Predictor)^40^. Hsa-miR-144 has 112 annotated disease associations, 36 of which with causality. Most of these were related to cancer and some instances include also neurodegenerative diseases (Supplementary Table S13).

***Functional context: MIENTURNET (MicroRNA Enrichment TURned NETwork***^42^) is a web-tool recently developed to analyse miRNA-gene targets interaction networks. In a first step the tool creates a list of significantly enriched interacting target-genes from a user-defined list of miRNAs, prioritizing the target genes based on a user-defined FDR cut-off. Following this, a network of miRNA-target interactions genes is constructed, to enable the exploration of the network properties of the miRNA-genes complex interactions.

The list of 419 miRNAs belonging to community 24 of dnPD, that includes hsa-miR-144-3p, was fed into the tool: Supplementary Tables S14 and S15 report all summary statistics concerning the corresponding pathways enrichment. Results, offering enrichment from KEGG and Reactome databases, present again a majority of cancer-related pathways, as well as, interestingly, platelets associated functions.

**Figure 4:**
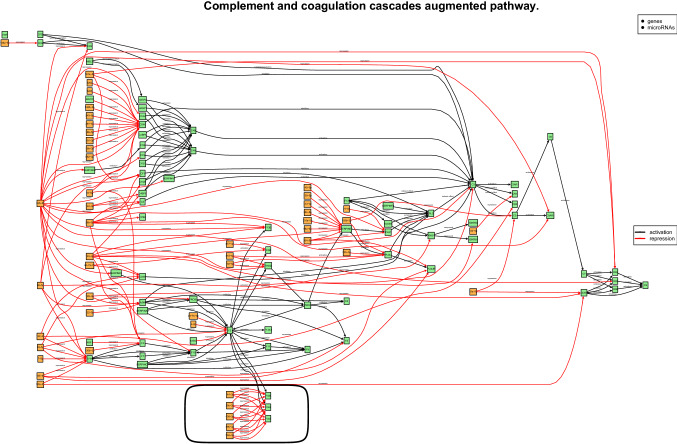
MiRIntegrator analysis for hsa-miR-144-3p. Graphical representation of the miRNA-augmented KEGG pathway hsa04610 “Complement and coagulation cascades” obtained using MiRIntegrator^67^ (R package version 1.22, http://datad.github.io/mirIntegrator/, and R software version 3.5.1). The repression activity of hsa-miR-144-3p to its targets *FGG*, *FGA* and *FGB* genes is highlighted with a black oval.

To gain additional insight in the above findings, we exploited MiRIntegrator^67^ an additional tool designed to visualize KEGG enriched pathways in the form of *miRNA-augmented pathways*, that is, the graphical integration of miRNAs of interest into signalling pathways, to highlight a pictorial representation of the miRNA-mRNA targets interactions. This integration in particular, highlights post-transcriptional gene repression mediated by the miRNAs of interest, giving additional causal insight into the phenotype under study (Fig. 4).

## Discussion

**Figure 5:**
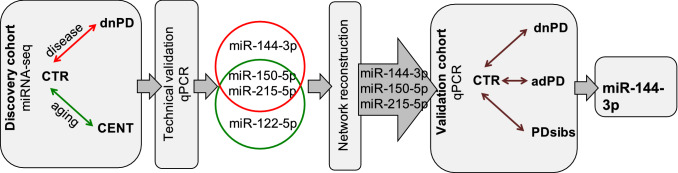
Summary of the overall experimental design and results pipeline. The figure was drawn using Microsoft Office 2016, Power Point. (https://www.microsoft.com/).

In the present work we explored the possibility to identify candidate-circulating blood biomarkers for PD within the (healthy) ageing framework. An overview of the obtained results, together with the methods applied, is summarized in Fig. 5.

First, we characterized by miRNA-seq the sera from a discovery cohort that includes dnPD, CTR and CENT. De novo PDs are a valuable model to study the disease as they represent the early stage of the malady and measurements are not affected by drug treatments.

Differential expression analysis led to the identification of a subset of modulated miRNAs selected for their relevance across different contrasts and from the literature. Among them, the technical validation by qPCR confirmed the differential expression of four miRNAs: hsa-miR-122-5p, hsa-miR-144-3p, hsa-miR-150-5p and miR-215-5p. While hsa-miR-122-5p was specific for the contrast CENT vs CTR and hsa-miR-144-3p was specific for the contrast dnPD vs CTR, hsa-miR-150-5p and miR-215-5p were similarly down-regulated in dnPD and CENT with respect to CTR. This observation was of potential interest because it supported our original hypothesis of a link between ageing and PD.

Accordingly, we observe that hsa-miR-150-5p and hsa-miR-215-5p, and noteworthy hsa-miR-122-5p and hsa-miR-144-3p, belong to the same miRNAs’ community identified via complex network modelling. Communities offer a first indication of the potential synergies or similarities shared by the nodes of a network and produce therefore an independent measure of the relevance of these miRNAs in the corresponding cohort, giving additional strength to the robustness of the finding of the differential analysis. Several examples exist in literature of application of complex networks to miRNA analyses. For instance, in Cantini et al.^51^ the authors proposed a pipeline to identify clusters of miRNAs cooperatively involved in triple negative breast cancer. Other works in the area also identify miRNA-target networks^52^ or miRNA-diseases networks^53^, to detect groups of miRNAs playing an important role in pathologies and diseases.

We then focused on the three miRNAs differentially expressed in PD (hsa-miR-144-3p, hsa-miR-150-5p and miR-215-5p) and assessed them in an independent cohort comprising dnPD, adPD and PDsibs. In this experimental context, hsa-miR-144-3p differential expression was confirmed for dnPD, offering a robust result for potential translation in preventive medicine, a fundamental tool in the fight against the disease. Additionally, hsa-miR-144-3p was also observed to be down-regulated in adPD and in a risk population such as PDsibs, in a statistically significant manner, but lacking of appropriate magnitude (Corrected p-value < 0.05 but log2(FoldChange) <|1|), granting further research in this direction to assess the relevance of this marker not only in prevention but also in the progression of the disease. It is worth noting that hsa-miR-144-3p did not change in centenarians that, despite the advanced age and the associated nigral neural loss^54^, did not develop clinically overt PD.

In our experimental settings, hsa-miR-144-3p did not change with age. Similarly, we did not find evidences in the literature supporting age-related differential expression in serum, with a single exception reporting an increase in mir144 (precursor of hsa-mir-144-3p) expression in cerebellum during ageing^55^. The same study also found an increase of mir144 in the frontal cortex of Alzheimer’s disease (AD) patients. One of the targets of hsa-mir144-3p is the Amyloid Precursor Protein (APP), a pivotal gene in AD, which also plays a role in regulating mitochondrial activity^56^. Accordingly, the authors demonstrated that mir144-3p was down-regulated in vitro and this resulted in induced mitochondrial dysfunction. The expression of mir144-3p was assessed also in brain tissue from PD patients, showing down-regulation in the pre-frontal cortex^57,58^, and upregulation in the cingulated gyrus^59^. Down-regulation is also confirmed in a recent comprehensive analysis of sncRNAs in blood^60^.

Finally, we used a variety of functional analysis tools to get insights into the role of hsa-mir144-3p in PD. Among the pathways that emerged as significant, those related to platelets functions are of particular interest.

Indeed, alterations in coagulation are among the known hallmarks of systemic inflammation and changes in the normal blood clotting have been described in PD^61^. Several studies demonstrated that platelets share molecular pathways with neurons and neuronal disorders^62^ and morphological and biochemical alterations were found in platelets from PD patients^63,64^. Furthermore, Fibrinogen Gamma Gene (FGG) is among hsa-mir144-3p targets and multiple reports show changes in FGG levels in PD patients’ plasma^65,66^.

Recently, Patil et al.^18^ evaluated miRNAs in serum from drug naïve PD samples and controls and identified three differentially expressed miRNAs (miR-335-5p, miR-3613-3p, miR-6865-3p). In our dataset, miR-6865-3p and miR-3613-3p had very low to no expression, while miR-335-5p was down-regulated both in CTR and PD versus centenarians. The expression level of this miRNA was not significantly different in dnPD with respect to CTR, therefore it was not selected for technical validation.

## Conclusions

Our results consistently show the down-regulation of hsa-mir144-3p in early PD, robustly confirmed across a variety of analytical and experimental analyses. Additionally, hsa-miR-144-3p was also observed to have a down-regulated trend (statistically significant, but lacking of appropriate magnitude) in adPD and in PDsibs, offering an important starting point for the investigation of this marker’s role in the progression of the disease. Functional analysis additionally revealed that hsa-miR-144-3p is involved in the regulation of coagulation, a process known to be altered in Parkinson’s Disease. These results suggest that hsa-miR-144-3p may be an early marker of PD. Further research is needed to unveil the functional details of its relevance and involvement to confirm these promising results.

## Supplementary Information

Supplementary Information 1.

Supplementary Information 2.

## Data Availability

The dataset supporting the conclusions of this article is available in the GEO repository (GSE180193).

## Abbreviations

PD: Parkinson’s Disease
miRNA: Micro RNA
NGS: Next generation sequencing
CENT: Centenarians
CTR: Controls
dnPD: Drug-naïve Parkinson’s Disease
adPD: Advanced Parkinson’s Disease
PDsibs: Parkinson’s Disease siblings
UMI: Unique molecular identifier
DEMs: Differentially expressed miRNAs
RQ: Relative quantification
MTIs: MiRNA–mRNA target interactions
FC: Fold change
