# Supplementary material for: Early downregulation of hsa-miR-144-3p in serum from drug-naïve Parkinson’s disease patients
Source: Sci Rep. 2022 Jan 25;12:1330. doi: 10.1038/s41598-022-05227-6 (PMC8789812; doi:10.1038/s41598-022-05227-6)
Supplement: Supplementary file 1 — Supplementary Information 1. [file 41598_2022_5227_MOESM1_ESM.docx]

**Additional information**

**Additional file 1:** The document contains Supplementary Tables 1-15. Table S1: Numbers of differentially expressed miRNAs (DEMs) obtained from different DESeq2 contrasts. Differential expression analysis was performed by comparing the different phenotypic groups of samples in the same collection site or by combining samples of different phenotypic groups or collection sites Centenarians were treated as both as super-controls, by grouping them together with the controls and comparing them with PD samples, and as test group (grouped with PD samples and compared with controls). Table S2: List of 28 miRNA targets selected for technical validation, with results of differential expression analysis. Nine miRNAs were selected among those detected as DE both in dnPD vs CTR+CENT and dnPD vs CTR contrasts, while other eight miRNAs were selected among those detected as DE both in contrasts specific of GOE Collection Site. Some miRNAs were selected among those detected as DE specifically in one contrast among dnPD vs CTR, dnPD vs CTR_GOE and dnPD vs CTR_GOE (MALES). Other targets have been selected from the intersections among at least 2 contrasts. Two targets emerged from the literature as involved in inflammation, named “inflamma-miR” (Caggiu et al. eNeurologicalSci 2018, Olivieri et al. Font. Genet. 2013). Table S3: Number of samples analysed for qPCR technical validation. Table S4: Results of qPCR analysis on dnPD samples vs controls, ranked by Corrected P-Value. Red text means that the qPCR result is statistically significant and Fold Change is above boundary. Table S5: Results of qPCR analysis on centenarians samples vs controls, ranked by Corrected P-Value. Red text means that the qPCR result is statistically significant and Fold Change is above boundary. Table S6: Results of qPCR analysis on dnPD samples vs centenarians, ranked by Corrected P-Value. Red text means that the qPCR result is statistically significant, and Fold Change is above boundary. Table S7: Number of nodes and edges of the miRNA networks reconstructed with ARACNE for each biological group of samples. Table S8.1: Weighted node degrees statistics of the distributions in the communities analysed. Table S8.2: Unweighted node degrees statistics of the distributions in the communities analysed. Table S9: Results of qPCR analysis on clinical dnPD samples vs controls, ranked by Corrected P-Value. Red text means that the qPCR result is statistically significant and Fold Change is above boundary. Table S10: Results of qPCR analysis on clinical adPD samples vs controls, ranked by Corrected P-Value. Red text means that the qPCR result is statistically significant and Fold Change is above boundary. Table S11: Results of qPCR analysis on clinical Pdsibs samples vs controls, ranked by Corrected P-Value. Red text means that the qPCR result is statistically significant, and Fold Change is above boundary. Table S12: Table of MirTarBase v7.0 MTIs of hsa-miR-144-3p. Table S13: Funcional annotation of the validated miRNAs with HMDD3.2 (database of miRNA-disease associations and of prediction of causal miRNAs for diseases). Table S14: Mienturnet functional enrichment results for microRNA target genes (KEGG database), ranked by ascending FDR. Referring to List 2 of the Mienturnet Experiments performed. Table S15: Mienturnet functional enrichment results for microRNA target genes (REACTOME database), ranked by ascending FDR. Referring to List 2 of the Mienturnet Experiments performed.
